# Animal source food eating habits of outpatients with antimicrobial resistance in Bukavu, D.R. Congo

**DOI:** 10.1186/s13756-021-00991-y

**Published:** 2021-08-26

**Authors:** Ghislain Bilamirwa Ngaruka, Brigitte Bora Neema, Theophile Kashosi Mitima, Antoine Sadiki Kishabongo, Olivier Basole Kashongwe

**Affiliations:** 1 Institut Supérieur Des Techniques Médicales (ISTM) de Bukavu, B.P. 3036, Bukavu, Democratic Republic of Congo; 2 Department of Laboratory Medicine, Provincial Referral General Hospital of Bukavu, Bukavu, Democratic Republic of Congo; 3 Livestock Production Systems Group, Department of Animal Sciences, Egerton University, Box 536-20115, Njoro, Kenya; 4 Leibniz Institute for Agricultural Engineering and Bioeconomy (ATB), Max-Eyth-Allee 100, 14469 Potsdam, Germany

**Keywords:** Antibiotic resistance, *Escherichia coli*, Animal Source Foods

## Abstract

**Background:**

Antibiotic resistance is a public health concern in Democratic Republic Congo and worldwide. It is usually caused by antibiotic over prescription or dispensing practices. The consumption of animal source food (ASF) could be another source of antibiotic resistance but is rarely studied. The objective of the study was to evaluate the eating habits of ASF by outpatients with antimicrobial resistance through an analysis of (i) the association of their antimicrobial resistance with ASF consumption; (ii) the influence of the types of ASF on their antimicrobial resistance.

**Methods:**

This is a retrospective analytical study conducted at three major Hospitals in Bukavu City (D. R. Congo). A total number of 210 patients, whose samples (mainly faeces and urine) had been subjected to bacterial examination, was included in this study. Morphological, biochemical and antibiotic susceptibility (using disc diffusion method) tests were performed on the samples. This served to isolate and identify resistant bacteria. Afterwards, patients responded to questions about the types and quantity of ASF eaten in the last week. We analysed data using descriptive statistics, logistic regression and non-parametric ranking tests.

**Results:**

*Escherichia coli* (37.1%), *Klebsiella pneumonae* (14.7%), and *Staphylococcus aureus* (13.8%) were the most prevalent bacteria. *E. coli* (68.4%) and *K. pneumonae* (87.5%) were multidrug resistant (MDR), while *S. aureus* (7.7%) was minor. Low beef (O.R. 0.737, C.I. 0.542–1.002) and pork (O.R. 0.743, C.I. 0.560 – 0.985) consumption led to significantly (*p* < 0.05) lower risks of resistance to ciprofloxacin. Patients eating three different ASF per week had the highest resistance score (20.67) and high consumption rates of goat meat, pork and milk (41.5%).

**Conclusion:**

The findings of this study suggest a contribution of human nutrition to antimicrobial resistance frequency. Our results show the existence of a high prevalence of multi-drug resistant bacteria in patients for which eating beef, pork and drinking milk are major risk factors. Therefore, a stricter control of antibiotic usage in livestock production and of their presence in ASF is recommended.

## Background

Bukavu is a fast-growing city in the South-Kivu Province, located in the East of the D.R. Congo. It was benefiting highly from the improvement of security in recent years. Strategically located at the shores of Lake Kivu, the city is supplied with Animal Source Food (ASF) from major local producing hubs as well as across the borders of Rwanda and Burundi [1]. The growing population coincides with increased ASF consumption, while production levels remain low within the country (Robinson and [2, 3]. This situation incentivise transboundary import of livestock and products to Bukavu, which quality and safety is not always ascertained. Three major local sources of ASF to Bukavu are existing, including Goma (Northern Kivu), Plaine de Rusizi (South Kivu), and Kavumu (South Kivu). Across borders, Bukavu is supplied with ASF from Kamembe (Rwanda), Burundi (via Uvira), and Uganda (via Goma or Kamembe). Imported products vary in quality and may pose a risk for consumers’ health and safety, if no proper control is applied. Indeed, food borne disease outbreaks including diarrhoea, cholera, and typhoid are increasing in number and severity in the Country [4, 5]. Globally, foodborne diseases are responsible for 1 of 3 deaths and diarrhoeal diseases account for 70% of total foodborne diseases mainly caused by *Salmonella* and *Escherichia coli.* Mahangaiko et al. [6] reported a contamination range of 11 to 27% of *Salmonella spp* along the meat chain in Kinshasa. Antimicrobial residues in ASF and associated development of resistance in people in D.R. Congo has not gained sufficient attention to shed light on the potential danger. Among the few available studies, Irenge et al. [7] reported a range of 20 to 100% resistance of *E. coli*, *Salmonella, Staphylococcus aureus* and *Coagulase Negative Staphylococci* isolates from patients to a wide range of antibiotics including those commonly used for livestock treatment: quinolones, sulfamides, tetracyclines and β-lactams. The developed resistances are often attributed to an uncontrolled use of antibiotics by patients and the transfer of acquired resistance to antimicrobials from agriculture to humans, which is more and more evident [8]. The European Union has developed a framework to control the use of anti-microbials in agriculture to prevent the transfer of resistances to people [9]. In Africa, specifically in West and East Africa, pathogenic microorganisms, pesticides residues and aflatoxins have gained attention recently in food safety regulations [10]. In the D.R. Congo, no specific approach is present to control the use of antimicrobials in agriculture. This could be directly associated with the consumption of ASF by patients admitted to major hospitals in Bukavu. We hypothesize that regular consumption of contaminated ASF from antibiotic-treated livestock could be associated with enhanced antibiotic resistance in patients in Bukavu town. We base this hypothesis on the potential causal relationship between consumption of ASF, foodborne disease and health status of households described by Randolph et al. [11].

## Methods

### Study setting

The study used a cross-sectional design to sample outpatients from 3 health zones (corresponding to 3 estates) of Bukavu city in D.R. Congo. Three hospitals, namely, Provincial Referral General Hospital of Bukavu, Referral General Hospital of Panzi and Biopharm Hospital were retained as study sites. The study was conducted between March and May 2019. We included outpatients sent by their practising physician to laboratories of the selected hospitals for antibiogram tests and interviewed them afterwards.

### Data collection and processing

During the months of March to May, a total of 210 outpatients who attended the selected hospitals were recruited for the study. The selection of the random sample of 210 patients followed recommendations to obtain an effective sample size of 15–20% of the total population under study (< 4000 patients visited the laboratory facilities of the selected hospitals for antibiogram test during the study period) with 95% confidence level and 0.05 precision level [43, 42].

After obtaining their informed consent, we interviewed patients about their meat and milk eating habits. This included the type of ASF (beef, chicken, goat, pork, and milk), the weekly frequency of consumption and the estimated quantity. The quantity, described as the number of portions, was then converted in grams and multiplied by the frequency of consumption to obtain the weekly ASF intake as described by Sanusi and Olurin [12]. This allowed us to categorise the weekly ASF consumption as low or high based on quantitative measures. Respondents eating less than 200 g of each ASF per week were considered as not regular consumers, while others were considered as frequent consumers, according to Bauer et al. [15]. We also collected data from patients’ socio-demographic characteristics, as well as bacteriological results from the laboratory analyses.

Laboratory tests practiced in the selected hospitals consisted of the isolation of causative organisms and in-vitro susceptibilities to several antibiotics. Samples were composed of urine (45%), faces (35%) and others, including blood and pus (20%). Isolation of causative microorganisms is routinely conducted using differential plating on specific selection media, including MacConkey Agar, EMB Agar, Baird Parker Agar, DTC Agar, Bile Esculin Agar and XLD Agar. Further morphological and biochemical tests, including Gram’s stain, oxidase, catalase, gas production, urease, motility, hemolysis and gelatinase test for each suspected organism were performed [14]. The disk diffusion method was used for antibiotics susceptibility test, following the procedure outlined by Bauer et al. [15] and updated by CLSI [16]. Briefly, bacterial isolates were inoculated in Mueller Hinton Agar plates before antibiotic disks were placed in the plates and incubation conducted for 24 h. Inhibition zone was then read using a measuring calliper [16]. The antibiotics tested included: ciprofloxacin (CIP); norfloxacin (NOR); gentamycin (GEN); amikacin (AMK); ceftriaxone-cefotaxime (CRO_CTX); ceftazidime (CAZ); cefuroxime (CXM); impinem (IPM); meropenem (MEM); tetracycline (TET); doxycycline (DOX); oxacyline (OXA); piperacillin (PIP); clarithromycin (CLR); erythromycin (ERY); clindamycin (CLI); chloramphenicol (CHL); and vancomycin (VAN). No invasive procedure was performed on the patients of this study, because we referred to the results of the above-mentioned laboratory tests.

### Statistical analysis

Data processed in MS Excel 2007 were analysed using descriptive and inferential statistics in SPSS version 22. Descriptive statistics were used first with frequencies and percentages to describe the socio-demographic data, as well as occurrence and antibiotic resistance of isolated microorganisms. We also used descriptive statistics to compute the multidrug resistance of bacterial isolates, on the basis of resistance to 3 or more antibiotic groups. Means and standard deviation were used to describe consumption trends of ASF. We used logistic regression to assess the association between ASF consumption and antibiotic resistance of respondents, for which we computed the Odds Ratio (O.R.) and Confidence Interval (C.I.). We set “high consumption” as the reference value (= 1). The Hosmer and Lemeshow test was used to assess the model fit at the significance level of 5%. We finally used non parametric ranking tests to determine the influence of the number of ASF eaten on the observed resistance to antibiotics. The sum of scores, mean scores and P-values (bold) are reported.

## Results

### Socio-demographic characteristics and ASF consumption of respondents

Mean age of respondents was 46 ± 11.5 years and female was the predominant sex (58%). More than half of respondents (59%) were from the commune of Ibanda while only 19% came from Bagira. The majority (98%) of respondent stated they had eaten ASF at least once in the past week, but only 38.3% had high ASF consumption (Table 1).

**Table 1:** Description of socio-demographic characteristics of respondents (n = 210)

Parameter	N	%
Sex		
Female	122	58.1
Male	88	41.9
Age		
18–22	59	28.1
23—31	48	22.0
32–40	34	16.2
> 40	53	25.8
Commune		
Bagira	41	19.0
Ibanda	124	59.0
Kadutu	45	21.4
High ASF consumption		
Beef	195 (67)	92.8 (33.7)
Chicken	190 (26)	90.0 (13.1)
Goat	165 (40)	78.5 (24.1)
Pork	128 (76)	61.0 (58.0)
Milk	203 (135)	96.6 (65.8)
Overall	881 (344)	98.0 (38.3)

**Fig. 1:**
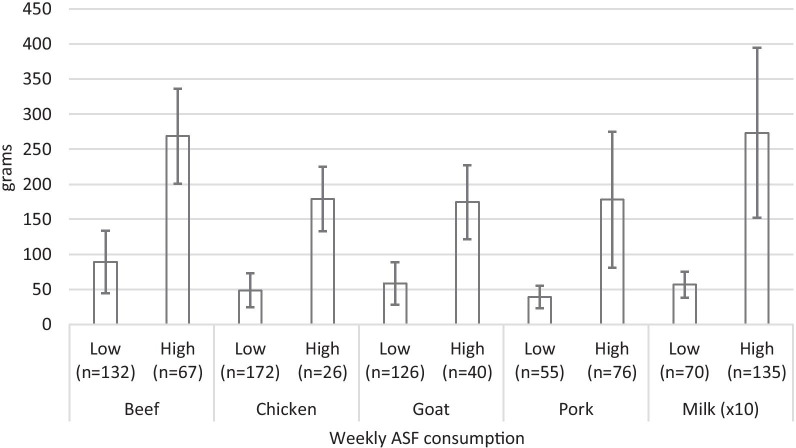
Consumption trends of animal Source Food (ASF) in Bukavu City. Milk consumption values are expressed in 10 × grams

Results summarizing consumption of animal -source -food (ASF) by respondents are presented in Fig. 1. The average consumption of beef and milk were above 250 g per person per week for high consumers, while low consumers ate between 50 to 100 g of ASF per week on average (Fig. 1).

Results of microbial cultures from patients’ samples are presented in Table 2. *Escherichia coli* (37.1%), *Klebsiella spp* (14.7%) and *S. aureus* (13.8%) were the most observed bacterial species. *Klebsiella oxytoca* (0.9%) and *Salmonella spp* (0.9%) were the least observed ones.

**Table 2:** Occurrence of bacteria in patients' samples

Bacteria	Isolates	
	n	%
*Escherichia coli*	43	37.1
*Klebsiella pneumonae*	17	14.7
*Staphylococcus aureus*	16	13.8
*Enterococcus spp*	11	9.5
*Enterobacter spp*	8	6.9
*Candida albicans*	7	6.0
*Morganella spp*	3	2.6
*Serratia marcescens*	3	2.6
*Pseudomonas spp*	2	1.7
*Cagulase Negative Staphylococcus*	2	1.7
*Streptococcus spp*	2	1.7
*Klebsiella oxytoca*	1	0.9
*Salmonella spp*	1	0.9
Total	116	100

Table 3 presents the results of the antibiotic resistance profiles of bacterial pathogens accounting for at least 2.5% of total isolates. The Findings indicate that *E.coli* isolates were resistant to most antibiotics tested, except amikacin, ciprofloxacin, chloramphenicol and tetracycline, for which less than 42% of samples showed resistance. Ciprofloxacine was the most tested antibiotic with 14.7% (90 tests).

**Table 3:** Antibiotic resistance profiles of major bacterial pathogens isolated in patients’ samples

Antibiotic	n (%)	*EC (%)*	*EB (%)*	*ECp (%)*	*KP (%)*	*MS (%)*	*SA (%)*	*SP (%)*	*SM (%)*
*Fluoroquinolones*									
CIP	90 (14.7)	41.9	50	81.8	61.5	66.7	26.7	0	100
NOR	35 (5.7)	61.5	NT	100	0	NT	100	100	66.7
*Aminoglycosides*									
GEN	64 (10.5)	58.3	57.1	100	62.5	100	28.6	0	0
AMK	39 (6.4)	30	0	0	11.1	0	50	0	0
*Cephalosporins*									
CRO_CTX	56 (9.2)	73.1	100	100	72.7	100	33.3	100	100
CAZ	31 (5.1)	100	100	100	100	100	NT	100	100
CXM	29 (4.7)	100	NT	NT	0	NT	0	NT	NT
*Carbapenems*									
IPM	39 (6.4)	0	NT	20	0	NT	0	NT	0
MEM	25 (4.1)	0	0	83.3	0	0	NT	0	0
*Tetracyclines*									
TET	23 (3.8)	33.3	NT	50	NT	NT	60	NT	100
DOX	16 (2.6)	50	100	NT	0	NT	0	NT	NT
*Penicillin*									
Augmentin	19 (3.1)	100	100	100	100	100	NT	NT	100
OXA	13 (2.1)	100	NT	NT	100	NT	83.3	NT	NT
PIP	12 (2.0)	100	NT	NT	100	100	NT	NT	NT
*Macrolides*									
CLR	18 (2.9)	62.5	75	NT	NT	NT	16.7	NT	NT
ERY	14 (2.3)	100	100	NT	0	NT	0	NT	NT
*Others (lincosamides, chloramphenicol and glycopeptides)*									
CLI	37 (6.1)	78.9	33.3	NT	100	NT	27.3	NT	NT
CHL	34 (5.6)	33.3	100	100	81.8	100	0	NT	100
VAN	16 (2.6)	100	NT	14.3	NT	NT	NT	NT	NT

*EC: E. coli; KP: K. pneumonae; SA: S. aureus; ECp: Enterococcus spp; EB: Enterobacter spp; MS: Morganella spp; SM: S. marcescens; SP: Salmonella spp*

*CIP: ciprofloxacin; NOR; norfloxacin; GEN: gentamycin; AMK: amikacine; CRO_CTX: ceftriaxone-cefotaxime; CAZ: ceftazidime; CXM: cefuroxime; IPM: impinem; MEM: meropenem; TET: tetracycline; DOX: doxycycline; OXA: oxacyline; PIP: piperacillin; CLR: clarithromycin; ERY: erythromycin; CLI: clindamycin; CHL: chloramphenicol; VAN: vancomycin. NT: Not tested*

Results in Table 4 present the antimicrobial resistance of the isolates from patients’ samples. We found that all Morganella spp, Pseudomonas spp and Serratia spp isolates were multidrug resistant as well as 68% of E. coli isolates. On the other hand, All C. albicans, K. oxytoca, Salmonella spp, CNS, Streptococcus spp isolates did not show multidrug resistance.

**Table 4:** Multidrug antimicrobial resistance (MDR) of the bacterial isolates from patients samples

Bacteria	Occurrence	Resistance (n)	MDR (n, %)	
*Escherichia coli*	43	38	26	68.42
*Enterobacter spp*	8	7	3	42.86
*Enterococcus spp*	11	8	2	25.00
*Klebsiella oxytoca*	1	1	0	0.00
*Klebsiella pneumonae*	17	16	14	87.50
*Morganella spp*	3	3	3	100.00
*Pseudomonas spp*	2	2	2	100.00
*Staphylococcus aureus*	16	13	1	7.69
*Salmonella spp*	1	1	0	0.00
*CNS*	2	1	0	0.00
*Serratia marcescens*	3	3	3	100.00
*Streptococcus spp*	2	1	0	0.00
*Candida albicans*	7	0	0	0.00

Table 5 presents the association between the detected antimicrobial resistances in patients and the ASF eaten, either high (Reference) or low as presented in Fig. 1. Patients eating less beef (O.R. 0.737; C.I. 0.542–1.002) and pork (O.R. 0.743; C.I. 0.560 – 0.985) had significantly (*p* < 0.05) lower odds of resistance to ciprofloxacin compared to those regularly eating. The odds of resistance to gentamicin was significantly influenced (*p* < 0.05) by milk consumption (O.R. 0.234; C.I. 0.059–0.924) in the univariable analysis. The odds of resistance to levofloxacin was significantly associated with beef (O.R. 0.690; C.I. 0.553 – 0.893) and chicken (O.R. 1.965; C.I. 1.370 – 2.820) consumption in the multivariable analysis.

**Table 5:** Associations between antibiotic resistance and consumption of ASF

ASF	Consumption level	n (%)	Univariable		Multivariable	
			O.R. (95% C.I.)	*P* value	O.R. (95% C.I.)	*P *value
*Ciprofloxacin*						
Beef	Low	132 (62.0)	0.875 (0.381 – 2.011)	0.753	0.737 (0.542–1.002)	**0.051**
	High	67 (31.5)	Reference		Reference	
Chicken	Low	172 (80.8)	1.689 (0.505 – 5.645)	0.395	1.414 (0.965 – 2.071)	0.076
	High	26 (12.2)	Reference		Reference	
Goat meat	Low	126 (59.2)	0.800 (0.304 – 2.106)	0.651	0.996 (0.716 – 1.385)	0.981
	High	40 (18.8)	Reference		Reference	
Pork	Low	55 (25.8)	0.378 (0.128 – 1.112)	0.077	0.743 (0.560 – 0.985)	**0.039**
	High	76 (35.7)	Reference		Reference	
Milk	Low	70 (32.9)	1.412 (0.528 – 3.774)	0.492	0.915 (0.680 – 1.230)	0.556
	High	135 (63.4)	Reference		Reference	
*Gentamicin*						
Beef	Low	19 (46.3)	0.921 (0.333 – 2.545)	0.874	0.839 (0.597 – 1.179)	0.311
	High	22 (53.7)	Reference		Reference	
Chicken	Low	35 (85.4)	3.214 (0.626 – 16.506)	0.162	1.176 (0.757 – 1.827)	0.470
	High	6 (14.6)	Reference		Reference	
Goat meat	Low	32 (78.0)	0.914 (0.236 – 3.539)	0.897	0.849 (0.569 – 1.266)	0.422
	High	9 (22.0)	Reference		Reference	
Pork	Low	21 (51.2)	1.705 (0.507 – 5.729)	0.389	0.954 (0.677 – 1.343)	0.787
	High	20 (48.8)	Reference		Reference	
Milk	Low	13 (31.7)	0.234 (0.059 – 0.924)	**0.038**	0.717 (0.499 – 1.030)	0.072
	High	28 (68.3)	Reference		Reference	
*Levofloxacin*						
Beef	Low	16 (50)	1.005 (0.355 – 2.843)	0.993	0.690 (0.533 – 0.893)	**0.005**
	High	16 (50)	Reference		Reference	
Chicken	Low	28 (87.5)	1.062 (0.286 – 3.945)	0.929	1.965 (1.370– 2.820)	**0.0001**
	High	4 (12.5)	Reference		Reference	
Goat meat	Low	25 (78.1)	2.133 (0.483 – 9.379)	0.316	1.293 (0.950 – 1.758)	0.102
	High	7 (21.9)	Reference		Reference	
Pork	Low	15 (46.9)	0.185 (0.033 – 1.020)	0.053	0.828 (0.648 – 1.058)	0.132
	High	17 (53.1)	Reference		Reference	
Milk	Low	10 (31.3)	3.294 (0.962 – 11.282)	0.058	1.257 (0.951 – 1.660)	0.108
	High	22 (68.8)	Reference		Reference	
*Ceftriaxone – cefotaxime*						
Beef	Low	18 (51.4)	1.905 (0.553 – 6.555)	0.307	1.190 (0.934 – 1.516)	0.160
	High	17 (48.6)	Reference		Reference	
Chicken	Low	33 (94.3)	2.059 (0.220 – 19.251)	0.527	0.899 (0.536 – 1.507)	0.685
	High	2 (5.7)	Reference		Reference	
Goat meat	Low	24 (68.6)	0.149 (0.033 – 0.667)	**0.013**	0.618 (0.471 – 0.812)	**0.001**
	High	11 (31.4)	Reference		Reference	
Pork	Low	16 (45.7)	0.265 (0.046 – 1.517)	0.136	0.808 (0.631 – 1.034)	0.091
	High	19 (54.3)	Reference		Reference	
Milk	Low	6 (17.1)	0.897 (0.160 – 5.023)	0.902	1.009 (0.726 – 1.401)	0.958
	High	29 (82.9)	Reference		Reference	

Reference: 1; OR: Odds Ratio; CI: Confidence Interval; Bold text is for statistical significance; P-value based on 0.05 Significance level

Results of antibiotic resistance scores based on the weekly quantity and types of ASF eaten are presented in Table 6. The majority of respondents (70.7%) ate at least two different ASF types in last week. Among the patients who reported eating only one ASF in the last week, 62% drunk milk, while the combinations of beef-milk (46%), goat-pork-milk (41.5%) and beef-chicken-goat-pork (50%) were mostly used in the other categories. The resistance score was highest for the combination of 3 ASF (score = 20.67), followed by the combination of two (19.67) and one ASF (18.89).

**Table 6:** Influence of the number of ASF frequently eaten on antibiotic resistance scores

Distinct types of ASF eaten	n (%)	ASF groups	Antimicrobial resistance				*P *value
			n	Within group %	Sum of Scores	Mean Scores	
0	7 (5.5)				2.22	0.32	0.05
1	30 (23.8)				18.89	0.63	
		Beef (B)	8	11.9			
		Goat meat (G)	4	6.0			
		Chicken (C)	2	3.0			
		Milk (M)	42	62.7			
		Pork (P)	11	16.4			
2	50 (39.7)				19.67	0.39	
		BM	29	46.0			
		BP	5	7.9			
		GM	5	7.9			
		GP	4	6.3			
		CM	6	9.5			
		CP	4	6.3			
		PM	10	15.9			
3	34 (27.0)						
		BGM	3	7.3	20.67	0.61	
		BCM	4	9.8			
		BCP	2	4.9			
		BPM	11	26.8			
		GCM	1	2.4			
		GPM	17	41.5			
		CPM	3	7.3			
4	5 (4.0)				1.56	0.31	
		BGPM	1	16.7			
		BCGP	3	50.0			
		CGPM	2	33.3			

## Discussion

The study investigated the question, whether antimicrobial resistance currently observed in patients of Bukavu City could be associated to the eating habits of ASFs. Recent studies report high levels of resistance to antimicrobials in patients visiting hospitals in the city [7, 17, 18, 40]. The increasing trend of resistance to antimicrobials in patients could be reasoned by the widely spread self-medication and the lack of clear governmental regulations for the use of antibiotics [17, 18, 40]. Additionally, there is growing concern about the contribution of food from animal origin on the observed resistances to antibiotics in humans. Indeed, evidence from other studies showed transmission of foodborne pathogens, similarities of relative frequencies and resistance genes of isolates to antibiotics between humans and animal isolates [36–39]. However, to date, there is scanty information in the literature on a possible role of animal source food as a cause of developed resistance in eastern D.R. Congo.

The current study identified that the majority of respondents regularly eat ASF, mostly milk (96%), beef (92.8%) and chicken (90%), but also goat meat (78.5%) and pork (61%). These ASF are part of the commonly kept livestock species in South Kivu Province, of which Bukavu is the capital city. Researchers reported that 85% of farmers keep livestock solely or in mixed systems [1]. They further indicated that farmers in South Kivu keep mostly chicken (70.5%), goats (66.1%), swine (46.4%) and cattle (19.6%) for sale and home consumption. This information corresponds with the responses from patients in our study, who mentioned eating chicken and goat meat more than pork. The higher carcass weight of cattle could explain the low percentage of kept units against its high consumption among the respondents in the current study.

The high percentages of kept livestock do not always reflect the quantity of ASF eaten at the household level. The D.R. Congo is among the least consumers of ASF in the world with 5.2 kg/capita/year for beef, 0.6 for milk and 0.1 for eggs [13]. In contrast, we observed in this study much higher ASF consumption in Bukavu than for the country average. Consumption of beef (13.97 kg/capita/year) and milk (14.21 kg/capita/year) for high consumers was comparable to some high ASF consumers in Sub-Saharan Africa. This includes the Republic of Congo, Benin and Kenya; although the latter has a much higher consumption of milk per capita (79.7 kg/year) [13]. Low consumers indicated about 4.6 kg/year for beef and 2.9 kg for milk, which is comparable to national levels. Chicken consumption was reported to be between 2.5 and 9.3 kg/capita/year, while it was between 2.0 and 9.2 kg/capita/year for pigs and between 3.0 and 9.1 for goat meat for low and high consumers, respectively. These reported consumption levels were generally higher than observed in Sub-Saharan Africa, except for poultry where low eaters are below the 6.7 kg/capita/year reported in Africa [2].

High and regular consumption of ASF from animals treated with antibiotics could pose a risk for humans to develop resistances [19], because the livestock industry uses a wide range of antibiotics also used in human medicine to fight various pathogens such as *E. coli, Salmonella* and *S. aureus* [20, 21, 39]. These authors describe ASF consumption as one of the routes for transmission of antibiotic resistance from animals to human, alongside with drinking contaminated water. The main groups of antibiotics used in livestock treatment include sulfonamides, sulfadiazine, lincosamides, tetracyclines, penicillin, betalactams, cephalosporins, quinolones, fluoroquinolones and macrolides/azalides aminoglycosides [22, 23].

We found that for *E. coli, Klebsiella spp, Enterobacter spp, Shigella spp* and *Salmonella spp* a high proportion of isolates showed resistance to certain antibiotics including cotrimoxazole, erythromycin and ampicillin, similar to what has been reported in Bukavu [17]. The most commonly isolated microorganisms in patients’ samples were *E. coli* (37.1%), *K. pneumonae* (14.7%), and *S. aureus* (13.8%). These microorganisms are among the most pathogenic in livestock production and reported multi-drug resistant among bacteria in ASF [39]. Köck et al. [39] reported the presence of livestock associated methicillin resistant *S. aureus* in patients’ samples, while Wu et al. [38] have reported a similar trend with resistant genes of *E. coli*. This finding is of high public health relevance because of the risk of human microbial pathogens gaining resistance to antimicrobials, especially in Sub-Saharan Africa. We found that *E. coli* isolates were resistant to most antibiotics tested, except Meropenem (100% susceptible), Imipenem (100% susceptible), and to some extent tetracycline (66.4% susceptible), chloramphenicol (66.4%), and amikacin (70% susceptible). Other researchers working on patients’ urinary tract infections [25], acute diarrhoea [26] and strains occurring in humans, animals or food [27, 28] reported also a resistance of *E. coli* to multiple drugs. Here, 68% of *E. coli* isolates were found to be multidrug resistant (to more than 3 antibiotic families). A similar trend was observed in Bukavu City by previous research that associated the observed multidrug resistance of several micro-organisms including *E.coli* [7, 32, 40]*.* They explained it with an irrational prescription and/ or use of antibiotics in the province. *Klebsiella pneumonae* and *Morganella spp, Pseudomonas,* and *Serratia spp* were also found to be multidrug resistant as reported in other studies [17, 33, 34]. These researchers associated the development of multidrug resistance to prior exposure to a group of antibiotics in the classes of fluoroquinolones, penicillin, glycopeptides, carbapenems and cephalosporins among other risk factors [17, 34, 35]. Isolates of *Salmonella spp* were found to be resistant to amoxicillin (88.3%), augmentin (95%), chloramphenicol (92%) cotrimoxazole (90%), doxycycline (73%) and negram (98%) [18]. The resistance of the bacterial isolates to antibiotics, although attributed to wrongful administration/uptake, could also be due to environmental contact, or consumption of food already contaminated by antibiotics [24].

We investigated the possible associations between resistances to antibiotics observed in microorganisms isolated from patients with their eating habits of animal source foods (ASF). The influence varied for different antibiotics and the type of ASF consumed. Resistance to ciprofloxacin was lower for patients eating less beef (0.875, CI: 0.381–2.011), goat meat (0.800, C.I: 0.304–2.106) and pork (0.378, CI 0.128–1.112) in the univariable analysis. In the multivariable analysis, only low pork consumption (O.R.: 0.743, CI 0.560 – 0.985) was found with significantly lower odds of resistance to ciprofloxacin than high consumption. Fluoroquinolones, the antibiotic family of ciprofloxacin, are widely used in monogastric livestock production. The common forms used are marbofloxacin, difloxacin and enrofloxacin. Ciprofloxacin is a major metabolite of the latter and studies have reported a high concentration of fluoroquinolones in the dust of pig barns (> 10 ng/mg) and in pork samples (315.3 µg/kg; 11.39 µg/kg and 27.02 µg/kg for ciprofloxacin, norfloxacin and ofloxacin respectively) [28, 29]. This could influence the transfer of resistant microbiota to consumers. In the same antibiotic family, our study reported a significant influence of beef (O.R. 0.69; CI 0.533 – 0.893) and chicken consumption (O.R. 1.965; C.I. 1.3970 – 2.820) on the risk of resistance to levofloxacin. This is in line with findings by Kyuchukova et al. [30], who reported a high concentration of levofloxacin (428 µg/kg) in chicken meat samples from the end of antibiotic treatment up to 8 days, although the concentration tends to decrease with time (56 µg/kg). These findings suggest a high risk to public health and add to the hypothesis that these food products could be causing resistance in humans if withdrawal periods are not observed. Indeed, resistance to antimicrobials can originate from sub-therapeutic and therapeutic use of antibiotic when withdrawal periods are not observed and, render patients’ treatment more complex.

Patients drinking less milk had significantly (*p* < 0.05) lower odds of resistance (0.234, CI 0.059–0.924) to gentamycin than those drinking high volumes. The difference was significant in the univariable but not in multivariable analysis. Gentamycin is commonly used in lactating dairy cows to treat intramammary infections. Previous studies show about 12.5% resistance of *S. aureus* isolated from milk to this antibiotic [31]. They further report 55.5% and 44.4% of milk samples containing residues of gentamycin 6 days after the treatment. This further explains the risk for consumers by animal-derived food, especially from the informal sector, where compliance with quality testing and withdrawal period are not observed.

We further investigated the possible influence of combining more than one ASF on the developed resistance to antibiotics. Results show that from eating 1 to 3 ASF the sum of scores for resistance to antibiotics increases. This could be explained by the influence of different ASF to different antibiotics, hence increasing the chance of resistance with the increase the number of ASF eaten during the same week. However, patients who reported eating four ASF during the same week had the lowest score. This could be due to the small number (only 5) of individuals in this category, not allowing to make a decisive inference. Hence, the risk for public health rises since the consumption of multiple antibiotic contaminated food may transmit multi-drug resistant bacteria to consumers. Marshall and Ley [19] explain that food from many different animal sources not only could contain high quantities of resistant bacteria but also point out the difficulty to attribute developed resistance in humans to the specific antibiotic. Hence, molecular techniques are able to confirm the identity of the microbial resistance gene in animals and humans [19]. In the context of Bukavu city, much remains to be done to understand the transmission pattern of antibiotic resistance from ASF to humans. An initial step would be the identification and quantification of microbial strains and antibiotic residues found in animal -source -food using molecular techniques.

## Conclusion

Antibiotic usage is described as the cause of bacterial resistance to antibiotics. This study has shown the existence of a high prevalence of resistant bacteria in patients for which frequent consumption of beef, pork, goat meat or milk were significant risk factors. The risk could be reduced by observing withdrawal periods before animals are sold and improving the surveillance of antibiotics use in livestock farms supplying the city.

## Data Availability

All data generated or analysed during this study are included in this published article (and its supplementary information files).
